# Unlocking the Potential of MBenes in Li/Na-Ion Batteries

**DOI:** 10.3390/molecules30132831

**Published:** 2025-07-01

**Authors:** Zixin Li, Yao Hu, Haihui Lan, Huicong Xia

**Affiliations:** 1 School of Materials Science and Engineering, Zhengzhou University, Zhengzhou 450001, China; 2 Materials Science and Engineering Program, Texas Materials Institute, The University of Texas at Austin, Austin, TX 78712, USA; 3 Department of Chemistry, Massachusetts Institute of Technology, Cambridge, MA 02139, USA; 4 Key Laboratory of Advanced Energy Materials Chemistry (Ministry of Education), Nankai University, Tianjin 300071, China

**Keywords:** Mbene, metal-ion batteries, two-dimensional materials, energy storage, electrode engineering

## Abstract

MBenes, an emerging family of two-dimensional transition metal boride materials, are gaining prominence in alkali metal-ion battery research owing to their distinctive stratified architecture, enhanced charge transport properties, and exceptional electrochemical durability. This analysis provides a comprehensive examination of morphological characteristics and fabrication protocols for MBenes, with particular focus on strategies for optimizing energy storage metrics through controlled adjustment of interlayer distance and tailored surface modifications. The discussion highlights these materials’ unique capability to host substantial alkali metal ions, translating to exceptional longevity during charge–discharge cycling and remarkable high-current performance in both lithium and sodium battery systems. Current obstacles to materials development are critically evaluated, encompassing precision control in nanoscale synthesis, reproducibility in large-scale production, enhancement of thermodynamic stability, and eco-friendly processing requirements. Prospective research pathways are proposed, including sustainable manufacturing innovations, atomic-level structural tailoring through computational modeling, and expansion into hybrid energy storage-conversion platforms. By integrating fundamental material science principles with practical engineering considerations, this work seeks to establish actionable frameworks for advancing MBene-based technologies toward next-generation electrochemical storage solutions with enhanced energy density and operational reliability.

## 1. Introduction

The rapidly growing demand for sustainable and efficient energy storage technologies has become one of the central challenges of the contemporary world [1,2]. As the global community shifts toward renewable energy sources such as solar and wind, which are intermittent by nature, robust energy storage systems are needed to stabilize power supply and maximize energy utilization [3,4,5]. Among various technologies, rechargeable batteries play a pivotal role due to their high energy density, scalability, and adaptability to diverse applications ranging from portable electronics and electric vehicles to grid-scale energy storage [6]. In this context, lithium-ion batteries (LIBs) have emerged as the dominant technology, owing to their exceptional performance in terms of energy density, cycle life, and efficiency [7,8]. However, concerns regarding Li scarcity, cost, and safety issues have motivated researchers to seek alternative battery chemistries, with sodium-ion batteries (SIBs) attracting substantial attention due to Na’s earth abundance and similar electrochemical properties [9,10]. Both LIBs and SIBs rely heavily on electrode materials capable of efficient ion intercalation or storage [11]. Layered materials have proven particularly effective in this regard because their unique structure facilitates ion diffusion and storage while maintaining structural integrity during charge–discharge cycles [12,13]. The past decade has witnessed remarkable advances in two-dimensional (2D) materials, which present high surface areas, tunable interlayer spacing, and plentiful active sites beneficial for electrochemical reactions [14,15].

Since graphene was first isolated, the family of 2D materials has continued to expand, now encompassing a variety of materials with unique physical and chemical properties, such as transition metal dichalcogenides (TMDCs), MXenes, carbon nitrides, hexagonal boron nitride (h-BN), black phosphorus, and phosphorene [5,16]. These materials demonstrate significant application potential in the field of energy storage, with the potential to drive innovations in battery and supercapacitor technologies to meet the growing demand for energy and the requirements of sustainable development (Table 1). Graphene’s high conductivity facilitates faster charging and discharging rates in batteries and supercapacitors. Its exceptionally large theoretical specific surface area provides abundant adsorption sites for ions, thereby enhancing the capacity of energy storage devices. TMDCs are a class of two-dimensional materials composed of transition metals (such as Mo, W, Ti, etc.) and chalcogen elements (S, Se, Te), featuring a layered structure with an appropriate interlayer distance that can accommodate the insertion and extraction of various ions [17]. The high specific surface area and abundant surface functional groups of MXenes confer significant advantages in the field of energy storage [18]. Carbon nitrides are a class of two-dimensional materials composed of carbon and nitrogen elements, exhibiting diverse structures and properties. Among these, graphitic carbon nitride (g-C_3_N_4_) is one of the most extensively studied. g-C_3_N_4_ possesses an appropriate band structure, making it a promising candidate for applications in photocatalysis and energy storage. h-BN has a layered structure similar to graphene, composed of alternating boron and nitrogen atoms. h-BN exhibits excellent thermal and chemical stability, with high thermal conductivity. Black phosphorus is an allotropic form of phosphorus with a layered structure [19]. When black phosphorus is exfoliated into single or few layers, it is referred to as phosphorene. Phosphorene has a direct bandgap and high carrier mobility, making it a promising candidate for applications in electronic devices and energy storage [20]. Among these, a novel class known as MBenes has recently emerged as a promising candidate for addressing key limitations associated with conventional electrode materials [21,22].

**Figure 1:**
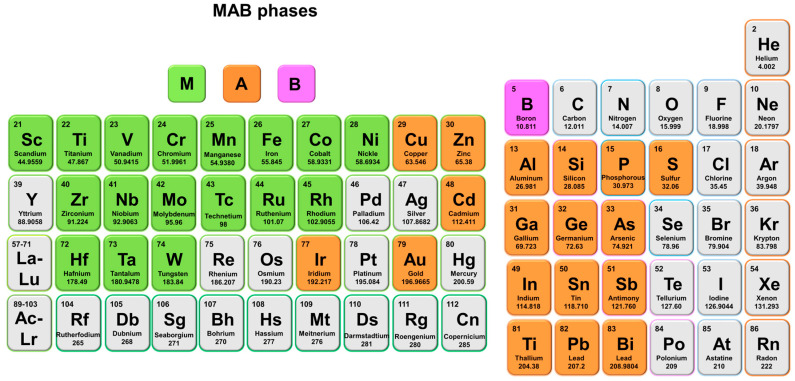
The variety of elements from the periodic table to be used to compose MAB phases.

MBenes are a relatively new family of 2D transition metal borides, analogous to yet distinct from the more widely studied MXenes, which are transition metal carbides or nitrides (Figure 1) [37,38]. While MXenes have gained significant traction in energy storage and conversion due to their metallic conductivity, hydrophilicity, and surface chemistry, MBenes offer unique advantages stemming from their boron-rich composition and structural features [39]. Generally formulated as M_n+1_B_n_, where M represents an early transition metal and B stands for boron, MBenes exhibit layered structures that combine high mechanical strength and excellent electrical conductivity with rich surface chemistry [40,41]. These characteristics make them particularly attractive for application as electrode materials in LIBs and SIBs. In LIBs, the MoB MBene synthesized by Xiong et al. maintained a reversible specific capacity of 144.2 mAh/g after 1000 cycles at a current density of 2.0 A/g when used as an anode, outperforming many reported MXene-based anode materials [42]. In SIBs, although the specific capacity of the original MBenes is relatively low, developing MBene-based heterostructures can effectively improve performance. The Mo_4/3_B_3_T_x_-MoS_2_@C composite anode prepared by Liu et al. demonstrated excellent rate capacity (340.6 mAh/g at 1.0 A/g) and durable cycling performance (267.2 mAh/g after 600 cycles at 2.0 A/g) in SIBs [43]. The unique performance exhibited in both battery systems positions MBenes as a promising key material for addressing current energy storage challenges, sparking researchers’ enthusiasm for further exploration.

**Figure 2:**
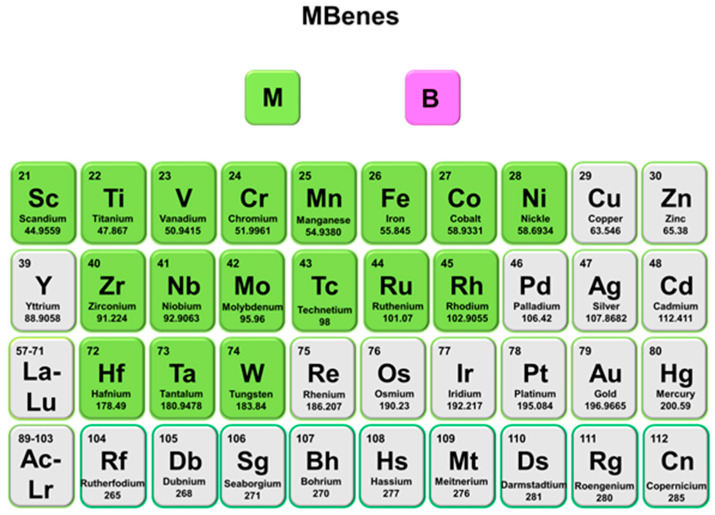
The variety of elements from the periodic table used to compose MBenes.

The conceptual birth of MBenes was closely linked to the exploration of MAX phases and their derivatives (Figure 2) [44]. MAX phases are ternary carbides or nitrides with layered hexagonal structures, featuring alternating layers of early transition metals (M), group A elements (typically from groups 13 or 14), and carbon or nitrogen (X) [45]. The chemical exfoliation of the A-layers from MAX phases results in MXenes, which retain the metal carbide/nitride layers but gain 2D morphology and active surface sites [46]. MBenes differ in that boron layers replace the carbon or nitrogen layers, yielding different bonding environments and electronic properties, thus opening new possibilities for optimizing electrochemical performance. MBenes have attracted intense research interest over the past few years due to their potential to overcome some of the intrinsic challenges faced by other 2D materials in battery applications [47]. These challenges include limited cycling stability, inadequate rate capabilities, and insufficient capacity retention under practical operating conditions. The boron-rich layers in MBenes confer enhanced structural rigidity and chemical stability, which help maintain electrode integrity during repeated ion intercalation and deintercalation. Moreover, the ability to tailor their surface chemistry through functionalization and doping enables optimization of their interaction with electrolyte ions, improving charge transfer kinetics and overall battery efficiency.

Early studies have demonstrated promising electrochemical behavior of MBenes when employed as anodes or cathodes in LIBs [48]. Their layered morphology promotes facile Li ion diffusion while providing abundant active sites, resulting in high specific capacities and enhanced rate performance. In SIBs, the relatively larger ionic radius of Na compared to Li introduces additional challenges, such as slower diffusion kinetics and larger volume changes during cycling. MBenes’ tunable interlayer spacing can accommodate these larger ions more effectively than some traditional materials, making them strong candidates for advancing SIBs technology. Despite these promising attributes, MBene research is still in its nascent stage, with many fundamental questions regarding their synthesis, structure–property relationships, and long-term cycling behavior yet to be fully explored [49]. The synthesis of high-quality MBene nanosheets with controlled thickness, defect density, and surface terminations remains a key challenge that directly impacts their electrochemical performance [50]. Additionally, understanding the precise mechanisms of ion storage—whether through intercalation, conversion, or pseudocapacitance—is critical for rationally designing MBene-based electrodes with superior durability and capacity [51]. Although MBenes theoretically exhibit excellent electrical conductivity and mechanical properties, these properties often fall short of expectations in practical experiments. This may be attributed to defects or impurities present during the preparation process [21]. A large number of defects can cause a sharp increase in the resistivity of the material, thereby weakening its conductive properties. This is inconsistent with the theoretically predicted high electrical conductivity, limiting its wide application in electronics and other fields [52].

**Figure 3:**
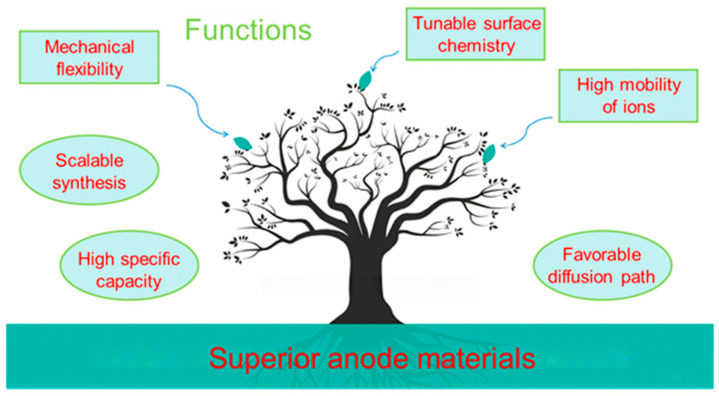
Schematic diagram illustrating the relationship among synthesis, structure, properties, and functions described in this review.

This review provides a comprehensive overview of MBenes, with a particular focus on their roles in LIBs and SIBs (Figure 3). First, we discuss the unique structural features and synthesis routes of MBenes, emphasizing recent advances in scalable and environmentally friendly production methods. Next, we analyze the physicochemical properties that contribute to their suitability as battery electrodes, including electrical conductivity, ion transport characteristics, and surface chemistry. Subsequently, we delve into the state-of-the-art applications of MBenes in Li and Na storage, covering material performance metrics, mechanistic insights, and the benefits of MBene-based composites. Finally, we present the current challenges faced by the field and outline prospective research directions that could unlock the full potential of MBenes for next-generation energy storage systems. By systematically reviewing the progress and prospects of MBenes in these two critically battery technologies, this work aims to underscore their transformative potential and promote further understanding and innovation in sustainable energy storage solutions.

## 2. Structure, Synthesis, and Physicochemical Properties of MBenes

With the rapid evolution of rechargeable battery technologies, the exploration of innovative electrode materials has become a central focus of materials science. MBenes—a relatively new group in the family of 2D compounds—have garnered widespread attention for their unique structures, versatile synthesis paths, and attractive physicochemical properties [53,54]. These factors combine to make MBenes strong candidates for advancing LIBs and SIBs technologies. This section outlines the fundamental structural characteristics of MBenes, reviews their principal synthesis approaches, and analyzes their core physicochemical attributes in the context of energy storage applications.

### 2.1. Structural Characteristics

**Figure 4:**
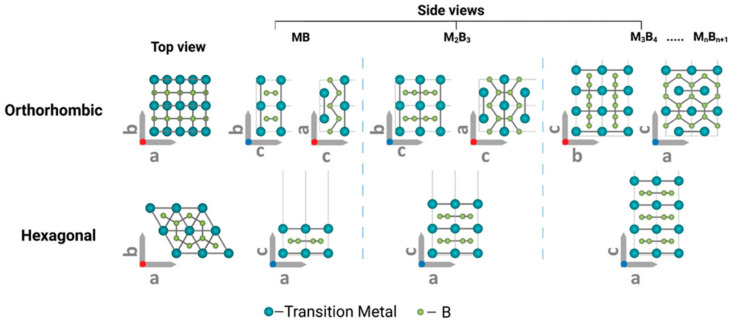
Schematic atomic arrangements predicted for typical MBenes [56]. Copyright 2022, Wiley-VCH.

MBenes are defined by the general chemical formula M_n+1_B_n_, where M is a transition metal (commonly Mo, Ti, V, Cr, Nb, etc.), B is boron, and ‘n’ is an integer (usually 1 or 2) (Figure 4) [55,56]. This results in a 2D layered structure; each layer comprises boron atoms sandwiched between layers of transition metal atoms. The strong in-plane metal–boron bonding and relatively weak out-of-plane van der Waals interactions enable these materials to exist as nanoscale sheets, reminiscent of the structural motifs found in other 2D materials such as graphene and MXenes.

Structurally, MBenes are closely related to MXenes, which have a general formula of M_n+1_X_n_, where X is carbon or nitrogen [57]. The primary distinction lies in the replacement of the carbon or nitrogen atoms with boron; this seemingly small substitution brings significant changes in both electronic structure and chemical reactivity. For instance, while MXenes inherit a combination of metallic conductivity, hydrophilicity, and certain catalytic activities from their parent MAX phases (M_n+1_AX_n_, where A is an ‘A-site’ element like Al or Si), MBenes generally exhibit higher theoretical electrical conductivities, greater mechanical robustness, and a broader range of electron-rich sites due to the boron’s unique electron configuration [58].

A key structural benefit of MBenes is their flexible and tunable interlayer spacing [59,60]. This interlayer distance can be precisely controlled by selecting transition metals, surface modifications, or post-synthetic treatments, making it possible to accommodate the varying sizes of guest ions—most notably, Na^+^ ions that are relatively large compared with Li^+^. This flexibility is an advantage over traditional layered materials such as graphite in Na ion systems, where the insufficient interlayer gap often prevents efficient Na^+^ intercalation.

In addition to their layered geometry and variable interlayer spacing, the surfaces of MBenes can be terminated by a variety of functional groups (e.g., –OH, –O, –F), introduced either during synthesis or via post-synthetic treatments [61,62]. These groups play a key role in tuning surface wettability, interfacial reactivity, and the chemical affinity toward ions or electrolytes. Furthermore, like all 2D materials, MBenes are susceptible to lattice defects, edge sites, and vacancies [63]. While excessive defects can negatively impact their long-term stability or electrical conductivity, controlled introduction of vacancies and edge dislocations can create new active sites for ion storage and enhance pseudocapacitive behavior.

### 2.2. Synthesis Methods

The synthesis of MBenes is foundational to unlocking their full technological promise. Reliable, scalable, and environmentally conscious production methods are required for both academic research and industrial-scale deployment. Several methods have been developed and refined in recent years, each offering unique advantages and posing specific challenges.

**Figure 5:**
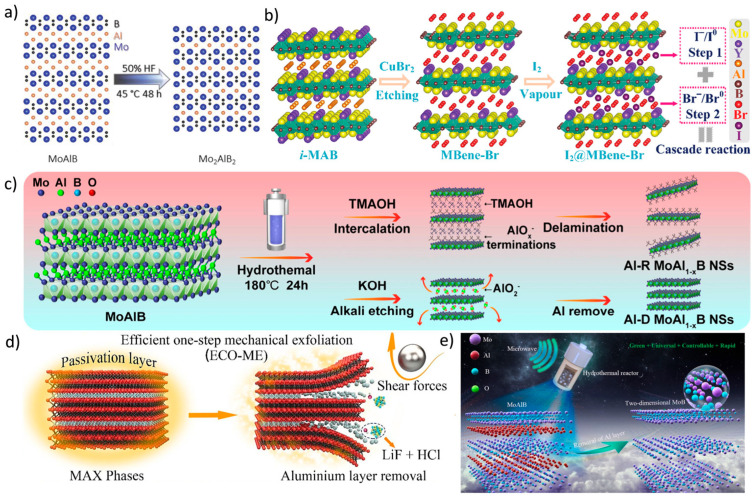
(**a**) Schematic illustration of the structural evolution from MoAlB to Mo_2_AlB_2_ [78]. Copyright 2023, Wiley-VCH. (**b**) Diagram showing the synthesis of I_2_@MBene-Br and the concept of a cascade reaction [79]. Copyright 2024, Wiley-VCH. (**c**) Illustration of the exfoliation method for Al-R and Al-D MoAl_1−x_B nanosheets in organic and strong alkaline solutions, respectively [80]. Copyright 2025, American Chemical Society. (**d**) Schematic depiction of the efficient one-step mechanical exfoliation (ECO-ME) synthesis pathway for MXenes [74]. Copyright 2023, Elsevier. (**e**) Illustration of the synthesis of 2D MoB MBenes using a microwave-assisted hydrothermal etching process in an alkaline solution [81]. Copyright 2025, Elsevier.

Traditional selective etching (de-A Method) (Figure 5a) [64,65,66,67] borrows from MXene synthesis. The classical route to MBenes involves selectively removing “A-site” elements from their MAX phase precursors (if such exist), typically using strong acids or molten salts. However, boron’s strong bonding with transition metals often demands more aggressive reaction conditions and careful control to avoid structural collapse or contamination.

Via chemical etching (Figure 5b) [68,69], many MBenes can be synthesized directly from binary transition metal borides by using halogen elements (e.g., Cl2 or Br2) or halide salts under high temperatures. This method is less reliant on MAX-like precursors and can yield high-purity products with adjustable thickness and lateral size. However, it often involves hazardous reagents, elevated temperatures, and precise post-processing to remove byproducts or terminate surfaces appropriately.

Topochemical modification and surface functionalization techniques (Figure 5c) [70,71,72,73], such as electrochemical treatment, can be used to modify surface chemistry, expand interlayer spacing, and introduce desired terminal groups. This approach is particularly valuable for optimizing ion transport properties or hydrophilicity for battery electrode applications.

Mechanical exfoliation (Figure 5d) [74] is similar to graphene production; MBenes can be mechanically exfoliated from layered bulk precursors by sonication or shear force. While this green and straightforward method avoids toxic reagents, its scalability is limited, and controlling the sheet thickness or size distribution remains challenging.

Regarding green and scalable synthesis (Figure 5e) [75,76,77], recent attention has focused on eco-friendly and scalable approaches such as low-temperature molten salt synthesis, hydrothermal processes, or even mechanochemical ball milling in the presence of benign solvents. These routes minimize hazardous waste and can be more easily upscaled while maintaining structural and functional diversity. As the field matures, integration of continuous flow reactors, modular process control, and recycling strategies are paving the way toward large-scale industrial production.

The chosen synthesis method deeply influences the MBene’s morphology, defect concentration, and surface terminations, and ultimately, its electrochemical performance in batteries. The future of MBene-based devices hinges on reproducible, safe, and cost-effective manufacturing.

### 2.3. Physicochemical Properties

The intrinsic physicochemical properties of MBenes provide the foundation for their rapid rise as next-generation battery materials. These features are pivotal to their superior performance in LIBs and SIBs.

Physical properties include electrical conductivity and ion transport [82,83,84]. Thanks to metallic bonding within the transition metal–boron sheets, MBenes boast excellent in-plane electrical conductivity—often surpassing that of MXenes and rivaling the best-known 2D materials. High electronic mobility ensures reduced internal resistance, enabling rapid charge/discharge capabilities and supporting fast ion transport at electrode/electrolyte interfaces.

With regard to specific surface area and layer spacing [85], the enlarged surface area and customizable interlayer distance provide abundant sites for ion adsorption and intercalation. This enhances reversible capacity and rate performance. Especially in the context of SIBs, where Na ions require larger galleries for migration, the MBene’s tunable structure provides a unique platform for efficient and rapid ion diffusion.

Surface chemistry and functional groups are relevant in this context [86]. The presence of polar functional groups (e.g., –OH, –O) further improves hydrophilicity, facilitating intimate contact with aqueous or organic electrolytes. These surface terminations can also participate in faradaic reactions, contributing to pseudocapacitive behavior and elevating the total storage capacity. At the same time, careful control of such chemical moieties is essential to optimize solid electrolyte interphase (SEI) formation, suppress side reactions, and extend cycle life—challenges especially pronounced during long-term cycling in lithium- and sodium-ion batteries.

With regard to their thermal and chemical stability [87], MBenes exhibit robust thermal and chemical stability under a broad range of operating conditions. The strong metal–boron lattice and well-defined surface terminations confer resistance to oxidation, hydrolysis, and other degradation pathways that often plague less stable layered materials. This durability is particularly advantageous for battery applications demanding high safety margins and long operational lifetimes.

The effects of defects should be considered [88,89,90]. While excessive defects (e.g., vacancies, grain boundaries) can compromise conductivity or structural integrity, a controlled amount of defects can create new redox-active sites, boosting capacity and sometimes even enhancing ion transport. Advanced MBene preparation and post-treatment methods aim to carefully balance these effects for optimal electrochemical outcomes.

In sum, the exceptional physicochemical profile of MBenes is centered on their electrical conductivity, adjustable layer structure, rich surface chemistry, and stability, and sets the stage for their powerful role in the next generation of LIBs and SIBs.

## 3. MBenes’ Applications in Lithium-Ion Batteries

The ongoing advancement of LIBs is essential to meeting the growing energy demands of modern technologies, from electric vehicles to renewable energy storage [91,92,93]. A primary focus of battery materials research is the development of novel electrode materials that offer higher capacity, enhanced cycling stability, and improved rate capability [94,95]. In recent years, MBenes, a family of 2D transition metal borides, have emerged as an innovative option for advancing LIB performance [96,97]. Their outstanding properties, including high electrical conductivity, robust mechanical strength, adjustable interlayer spacing, and rich surface chemistry, have positioned MBenes as promising candidates to overcome the limitations of conventional materials such as graphite or metal oxides. This section provides a comprehensive overview of the application potential of MBenes in LIBs, discussing their role as electrode materials, the impact of composites, and the complex Li storage mechanisms involved.

### 3.1. MBene as Electrode Materials: Capacity, Stability and Performance

Among the most appealing features of MBenes are their superior theoretical capacities. While graphite, the archetypal anode in commercial LIBs, is limited to a theoretical capacity of 372 mAh/g, various MBene compositions have demonstrated much higher specific capacities [98,99]. This arises primarily from their unique layered structures. MBenes typically adopt the M_n+1_B_n_ formula, where transition metal and boron layers alternate, yielding numerous accessible sites for Li ion intercalation [56]. Some experimental reports have indicated that MBene-based anodes can attain reversible capacities exceeding 500 mAh/g, with studies on Mo_2_B_2_, Ti_2_B_2_, and related MBenes revealing both high capacity and excellent reversibility over hundreds of cycles [100].

**Figure 6:**
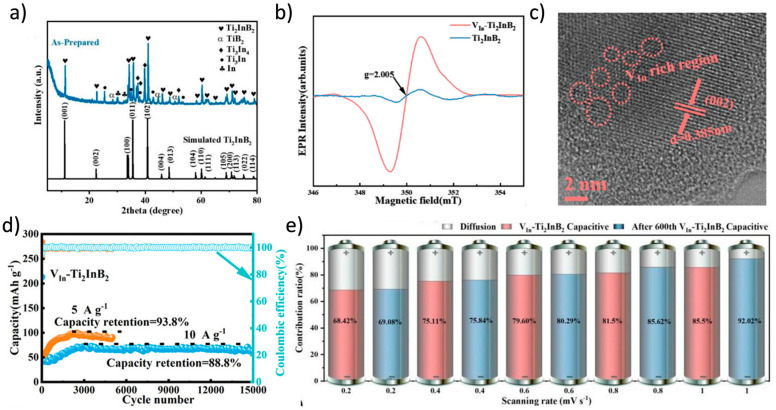
(**a**) XRD patterns and (**b**) EPR spectra of as-prepared Ti_2_InB_2_. (**c**) HRTEM image of VIn-Ti_2_InB_2_. (**d**) Long-term cycling performance of V_In_-Ti_2_InB_2_ electrodes tested at current densities of 5 and 10 A/g. (**e**) Capacitive contribution ratios of the V_In_-Ti_2_InB_2_ electrode at various rates, measured before and after 600 cycles [101]. Copyright 2024, Wiley-VCH.

**Figure 7:**
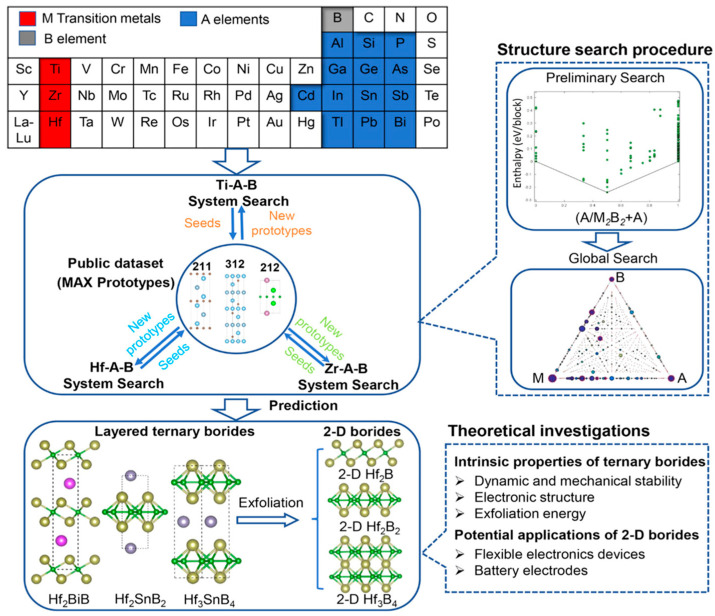
Computational strategy for discovering ternary borides. Each search for ternary compound structures involves two stages—a preliminary pseudo-binary structure search followed by a global ternary structure search. Theoretical analyses were conducted on each predicted layered ternary compound and its corresponding 2D structures [102]. Copyright 2020, American Chemical Society.

**Figure 8:**
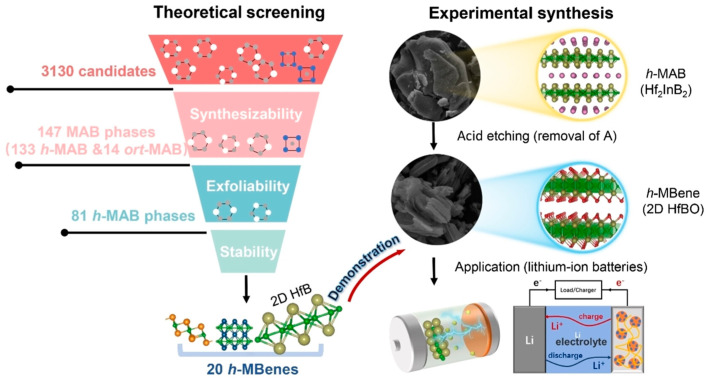
Overview of the calculation-driven approach used to discover *h*-MAB phases and h-MBenes, combining extensive high-throughput computational screening with experimental validation [52]. Copyright 2023, Wiley-VCH.

Theoretical studies predict reversible Li storage capacities for Ti_2_B_2_ up to 456 mAh/g [25], approaching or surpassing the theoretical maximum of graphite. Wang et al. employed a simple and effective ball milling method to introduce functional indium (In) vacancies into the *h*-MAB phase Ti_2_InB_2_ (Figure 6) [101]. The In-deficient Ti_2_InB_2_ *h*-MAB phase (V_In_-Ti_2_InB_2_), used as an anode material for LIBs, achieved a high capacity (600 mAh/g) and excellent long-term cycling stability. Compared to recently reported MAX/MAB phase materials, the V_In_-Ti_2_InB_2_ electrode in this study stands out in terms of reversible capacity and cycling stability across different current densities. Furthermore, through a combination of theoretical calculations and experimental tests, the researchers proposed a dual redox reaction Li storage mechanism for V_In_-Ti_2_InB_2_, involving the Li-In alloying reaction facilitated by indium vacancies on the (010) and (001) surfaces of V_In_-Ti_2_InB_2_, and the redox reaction of Li-TiB occurring at the (010) edge and (001) surface of V_In_-Ti_2_InB_2_. Research has found that not only can Ti_2_InB_2_-like configurations stably exist in the Zr-A-B and Hf-A-B systems, but two new configurations, “211” and “314”, have also been discovered (Figure 7) [102]. Notably, the identification of Hf_2_PbB and Hf_2_BiB structures provides a potential pathway for extending traditional MAX phases toward borides. High-precision theoretical calculations indicate that ZrB monolayers are excellent candidate materials for Li-ion battery electrodes. In addition, Wang and colleagues combined materials genome prediction with experimental validation to discover a series of novel hexagonal ternary MAB phases and their two-dimensional derivatives, hexagonal MBenes (Figure 8) [52]. Theoretical calculations revealed that the hexagonal crystal system ternary MAB phases (*h*-MAB) served as superior precursors for MBenes compared with the conventional orthorhombic crystal system MAB phases (ort-MAB). Based on theoretical predictions, the authors successfully synthesized three representative structural prototype materials of the h-MAB phase (Hf_2_InB_2_, V_3_PB_4_, and Hf_2_PbB). Taking *h*-MAB Hf_2_InB_2_as an example, they selectively etched away the In layer to successfully prepare the first hexagonal MBene (*h*-MBene), namely HfBO. Further application studies found that the 2D HfBO material showed great potential as an anode for LIBs.

The metallic nature of MBenes further supports rapid electron transfer during charging and discharging, contributing to their pronounced rate capability. Notably, many MBenes exhibit significant capacity retention even at high current densities, a property essential for fast-charging applications [79,103,104]. This combination of electrical conductivity and structural resilience sets MBenes apart from many emerging materials that suffer from slow kinetics or dramatic fading of capacity under demanding conditions. Cycling stability, a persistent challenge for many high-capacity electrodes, is another area where MBenes excel [89,105]. Owing to the robust covalent bonding within the metal–boron lattice, MBenes can endure the strain associated with Li insertion and extraction without undergoing the mechanical degradation that plagues materials like silicon or some metal oxides. Their planar and flexible nature further allows for minor volume changes during cycling, reducing the risk of cracking or pulverization. Consequently, MBene-based LIBs often demonstrate high Coulombic efficiencies and capacity retention above 90% after several hundred cycles.

**Figure 9:**
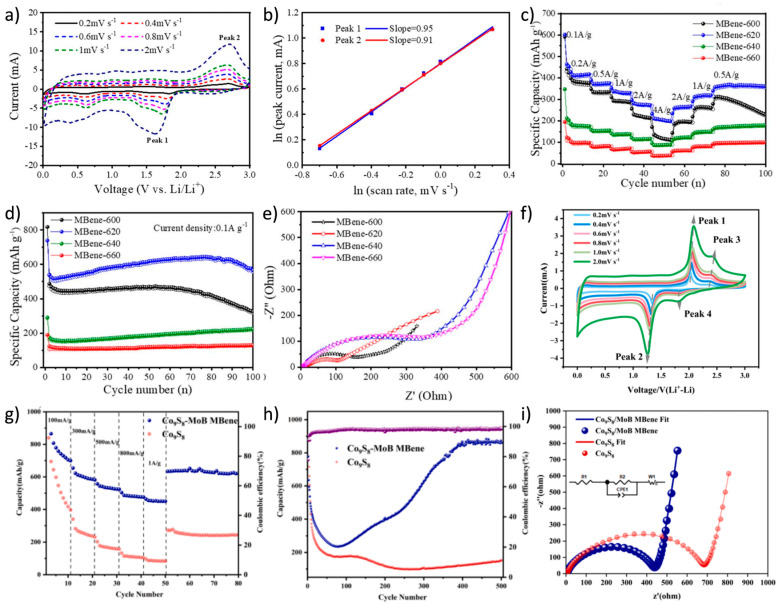
(**a**) CV curves of MBene-620 with the gradually increasing scanning rate. (**b**) Fitting diagram of relationship between ln (peak current) and ln (scan rate). (**c**) Rate capability of MBenes prepared at different temperatures, (**d**) cycle performance, and (**e**) EIS spectra of MBene anodes in LIBs [106]. Copyright 2024. The Royal Society of Chemistry. (**f**) CV curves of Co_9_S_8_-MoB MBene electrode at different scan rates (0.2–2.0 mV/s). (**g**) Rate performance, (**h**) cyclic specific capacity profiles at a current density of 300 mA/g, and (**i**) EIS of Co_9_S_8_-MoB MBene and Co_9_S_8_ electrodes in LIBs [107]. Copyright 2025. Elsevier Inc.

Identifying novel anode materials with high energy density, excellent cycle stability, and outstanding rate performance has become a key research direction in LIBs development. Chen et al. [106] successfully prepared Mo_x_B_y_ (MBene) derivatives with an accordion-like structure by completely etching the Al layer in MoAlB using the molten salt method. The b values for Peak 1 and Peak 2 were calculated to be 0.95 and 0.91, respectively, indicating that the energy storage kinetics of the MBene anode were dominated by a pseudocapacitive mechanism (Figure 9a,b). When used as an advanced anode material for LIBs, this MBene exhibited outstanding electrochemical performance, achieving a reversible specific capacity of 638.2 mAh/g after 100 cycles at a current density of 0.1 A/g (Figure 9c–e). Wang et al. [107] successfully synthesized a novel Co_9_S_8_-MoB MBene heterostructure material. The Co_9_S_8_-MoB MBene electrode exhibited outstanding performance, characterized by significant pseudocapacitive behavior. It maintained a specific capacity of 756.34 mAh/g after 200 cycles at a current density of 100 mA/g, with a cycle retention rate as high as 91.27%. The excellent cycle stability and rate performance were attributed to the two-dimensional layered structure of the conductive MoB MBene (Figure 9f–i).

### 3.2. MBene-Based Composite Systems: Synergy and Engineering

Despite their outstanding intrinsic properties, pure MBene electrodes are not immune to practical challenges, such as tendencies toward aggregation, restacking of nanosheets, and limited first-cycle efficiency due to SEI formation [108,109]. To mitigate these issues and enhance performance further, researchers have developed a range of composite electrode architectures that harness the synergistic effects of MBene with other functional materials.

Integrating MBenes with conductive carbon materials like graphene, carbon nanotubes, or amorphous carbon, carbon composites [110,111,112] benefit from enhanced electronic connectivity, flexibility, and dispersion. The carbon matrix acts to prevent restacking, thus preserving the accessible surface area for Li storage and facilitating fast charge–discharge kinetics. Hybrid structures such as MBene-graphene [113] or MBene-CNT [114] electrodes have been shown to achieve not only higher capacities but also better rate capability and cycling stability compared with pure MBene electrodes.

MBenes can also be combined with electroactive polymers such as polyaniline or PEDOT, as well as with redox-active metal oxides [115,116]. Polymers provide additional mechanical resilience and can buffer electrode expansion during cycling, while metal oxides introduce extra Li storage sites and support hybrid storage mechanisms. These composite designs effectively improve electrode robustness, enhance active material utilization, and suppress unwanted side reactions at the interface, collectively resulting in prolonged cycle life and stable operation under demanding charge–discharge regimes.

Structural design is very important [117,118,119,120,121,122]. The success of composite approaches relies heavily on precise control over composition, architecture, and nanoscale interface. Recent advances include layer-by-layer assembly, solution-phase co-precipitation, and in-situ hybridization methods that produce electrodes with optimized porosity, hierarchical order, and integrated conductive pathways. Smart design of MBene-based hybrids thus holds the key to balancing high capacity, mechanical integrity, and electrochemical stability in high-performance LIBs.

### 3.3. Lithium Storage Mechanisms in MBene Electrodes

Understanding the Li storage mechanism in MBene electrodes is crucial for unlocking their full potential and guiding rational material and device design. These mechanisms encompass both traditional intercalation and more complex surface and conversion reactions, the balance of which can be tuned by composition and surface engineering. In LIBs, the energy storage mechanism of MBenes primarily involves efficient Li^+^ intercalation/deintercalation, combined with some surface capacitive behavior. Its high conductivity and open layered structure allow for rapid Li^+^ diffusion, while changes in transition metal oxidation states (e.g., Mo^2+^/Mo^4+^) contribute to redox capacity. Compared with graphite, MBenes have larger interlayer spacing, reducing Li^+^ insertion resistance, and surface defects or functional groups may provide additional active sites, improving rate performance and cycle stability.

With regard to intercalation [123,124], the principal Li storage pathway in most MBene electrodes is the reversible insertion of Li ions between their 2D layers. This process minimally disrupts the MBene framework due to its robust metal–boron bonding and adjustable interlayer spacing. In situ X-ray diffraction and electron microscopy studies provide direct evidence that Li intercalation in many MBenes causes uniform, reversible lattice expansion that is distinctly less destructive than the phase transformations observed in alloying materials. Because this intercalation is highly reversible, MBene electrodes show good long-term capacity retention and cycling stability.

The surface chemistry of MBenes, including surface adsorption and pseudocapacitance, significantly impacts their storage behavior [125,126]. The presence of terminal groups like –OH, –O, or –F on MBene surfaces offers abundant sites for Li adsorption, contributing to a pseudocapacitive storage mechanism. Pseudocapacitance enhances high-rate capability as it involves rapid, surface-driven Faradaic reactions as opposed to slow bulk diffusion. Through careful tuning of surface functionalization, researchers can tailor the relative contributions of intercalation and pseudocapacitive processes, optimizing both energy and power densities.

Conversion reactions are also relevant [127,128,129]. For certain MBene compositions, particularly those containing multi-valent transition metals, Li may participate in partial conversion reactions involving temporary phase changes or redox-driven alloying. While these mechanisms offer opportunities for even higher capacities, they require careful management to avoid irreversible structural changes or capacity fade.

SEI formation and interface stability should also be considered [130,131]. Formation and control of the SEI is a vital consideration for all high-performance LIB electrodes, including MBene-based ones. The nature of MBene surfaces and their interactions with the electrolyte strongly influence SEI composition, uniformity, and stability, which in turn affect initial Coulombic efficiency, cycle life, and safety. Recent research suggests that surface-functionalized MBenes can promote formation of stable, compliant SEI layers that resist ongoing decomposition while maintaining efficient Li transport.

In summary, MBenes herald a new era of electrode material for LIBs, owing to their remarkable intrinsic properties, adaptable structures, and chemical versatility. Their high theoretical capacity, excellent rate performance, and robust cycling stability position them as promising candidates for advancing LIB technology beyond the limits of conventional electrodes. The development of MBene-based composites and hybrid architectures further expands design possibilities, allowing for the customization of electrode systems to meet diverse performance requirements. Ongoing research to elucidate the dynamic, multi-mechanistic storage behavior of MBenes, with a focus on optimizing capacity, stability, and safety. As synthetic methods advance and understanding deepens, MBenes are set to play a pivotal role in the next generation of high-performance, durable, and safe LIBs.

## 4. MBenes Applications in Sodium-Ion Batteries

SIBs have emerged as strong contenders in the race for next-generation energy storage systems, primarily due to Na’s abundance, low cost, and environmental compatibility [132,133,134]. However, the successful development of SIBs has been hampered by several technical challenges, chief among them being the larger ionic radius of Na compared with Li and the resulting difficulty in identifying electrode materials with compatible structures [135]. In this context, MBenes—a new class of 2D boride materials—offer remarkable promise, thanks to their tunable interlayer spacing, exceptional conductivity, and robust mechanical framework [136]. This section explores the structural advantages of MBenes for SIBs, provides a critical comparison with other anode materials, delves into Na storage and migration mechanisms, and highlights the considerable performance enhancements achieved through strategic use of composite materials.

### 4.1. Structural Advantages of MBene for Accommodating Larger Sodium Ions

One of the defining hurdles in SIB technology is the accommodation of Na ions, which possess a substantially larger ionic radius (1.02 Å) compared with that of Li^+^ (0.76 Å) [137]. This size difference leads to sluggish Na ion diffusion and significant structural deformation or even collapse in many conventional layered electrode materials [138]. Most traditional anode materials used in LIBs—such as graphite—are poorly suited to storing Na ions in their host structures, as the narrow interlayer spacing and insufficient flexibility restrict the insertion of Na ions and result in poor reversibility [139].

**Figure 10:**
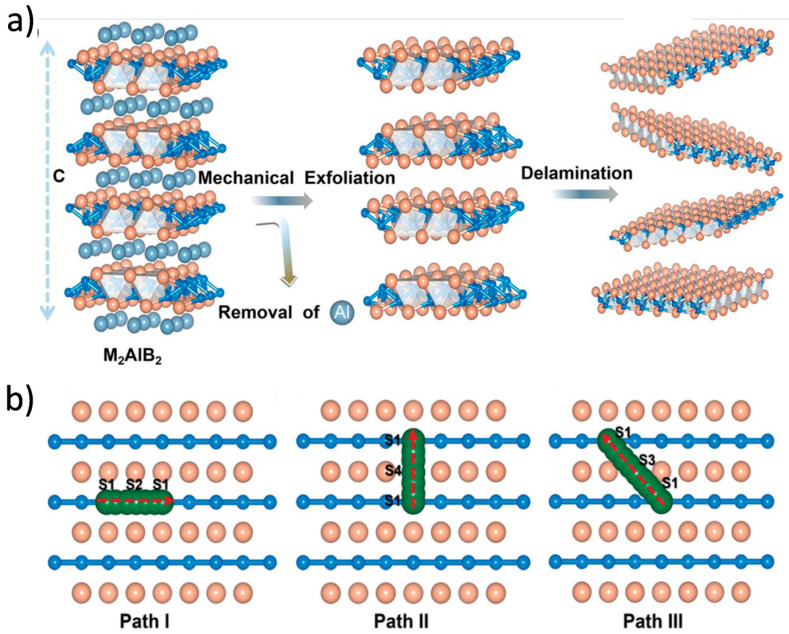
(**a**) Schematic illustration of Al removal to create MBenes through mechanical exfoliation of the MAB phase. (**b**) Diagram showing the metal cation diffusion pathways on monolayer MBenes, including S1 → S2 → S1, S1 → S4 → S1, and S1 → S3 → S1 routes [144]. Copyright 2019, Royal Society of Chemistry.

**Figure 11:**
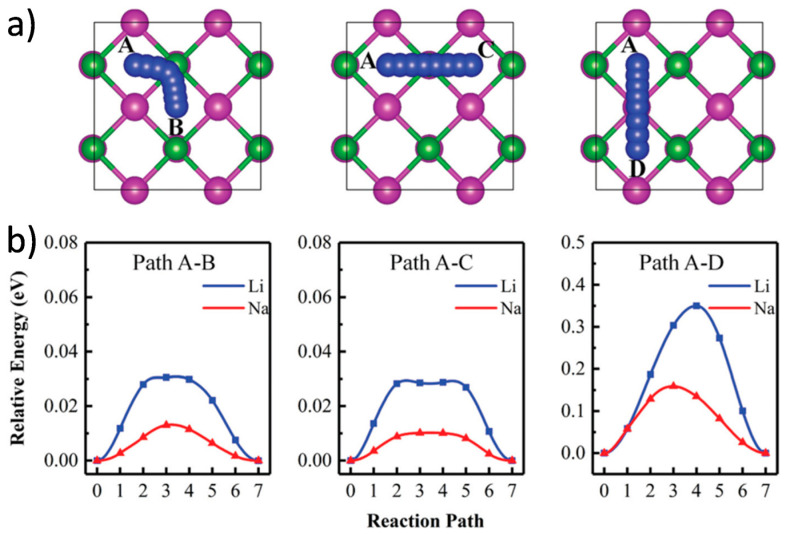
(**a**) Top-down views of three diffusion pathways for Li/Na on the tetr-Mo_2_B_2_ monolayer. Mo, B, and Li/Na atoms are shown in violet, green, and blue, respectively. (**b**) Diffusion energy barriers for Li and Na on tetr-Mo_2_B_2_ [145]. Copyright 2019, Royal Society of Chemistry.

MBene materials, characterized by their M_n+1_B_n_ composition and 2D morphology, inherently offer more adaptable and tunable interlayer spacing than many oxide or carbonaceous materials [140,141,142]. The strong yet flexible metal–boron framework of MBenes not only stabilizes their structure during repeated ion insertion and extraction but also allows controlled expansion of the interlayer space, which is critical for accommodating the bulkier Na ions. Surface chemistry modifications, including the introduction of polar functional groups or the intercalation of organic/inorganic spacers, are often employed to further increase gallery height and improve ion accessibility [143]. Gao et al. presented a series of orthogonal 2D MBenes based on first-principles density functional theory, which were obtained by mechanically exfoliating an MBene layer from a bulk MAB phase (Figure 10) [144]. These included V_2_B_2_, Cr_2_B_2_, Mn_2_B_2_, Ti_2_B_2_, Zr_2_B_2_, and Nb_2_B_2_. The thermodynamic and kinetic stability of monolayer MBenes at room temperature was confirmed through AIMD simulations and phonon spectrum analysis. In additionally, Wang et al. reported two novel Mo_2_B_2_ monolayers and investigated their stability, electronic structures, lattice dynamics, and potential as anode materials for energy storage, using crystal structure prediction techniques and first-principles calculations (Figure 11) [145]. The calculated phonon spectra and electronic structures revealed that the predicted tetragonal and trigonal Mo_2_B_2_ monolayers possessed excellent electronic conductivity and strong stability.

**Figure 12:**
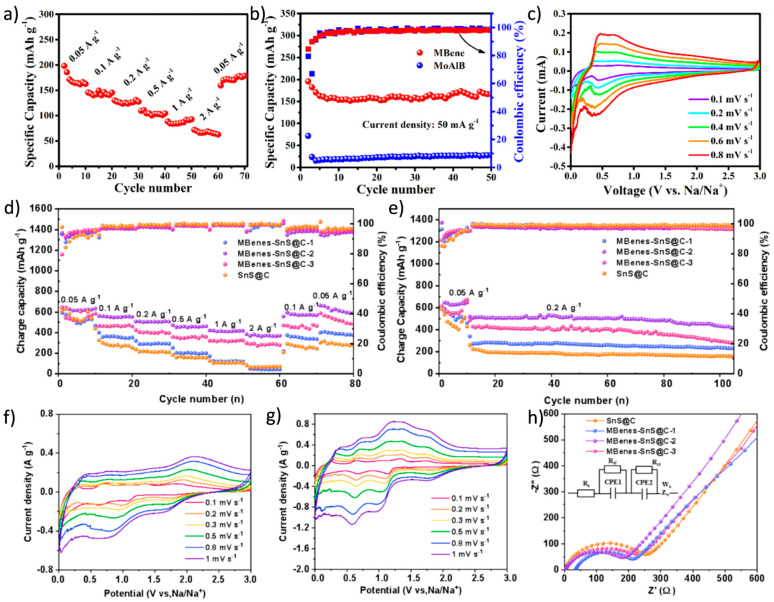
(**a**) Specific capacities of MoB MBenes at different current densities. (**b**) The cycle performance of MoAlB and MoB MBenes at 50 mA/g. (**c**) CV profiles at different scan rates of MoB MBenes [81]. Copyright 2025. Elsevier Ltd. (**d**) Rate performance and (**e**) cyclic performance of SnS@C and MBenes-SnS@C in SIBs. CV profiles of (**f**) SnS@C and (**g**) MBenes-SnS@C-2 at scanning rates of 0.1–1 mV/s. (**h**) EIS of SnS@C and MBenes-SnS@C [146]. Copyright 2025. Elsevier Inc.

Recent studies have shown that MBenes’ unique atomic configurations can effectively mitigate common degradation mechanisms such as exfoliation, restacking, and lattice collapse, which are factors that otherwise compromise SIBs’ long-term cycling performance [21]. The combination of high mechanical rigidity and structural tunability makes MBenes especially attractive not only for Na ion storage but also for sustaining stable operation over hundreds or thousands of cycles. Xiong et al. [81] proposed an efficient microwave-assisted hydrothermal alkaline solution etching strategy to peel off the MoAlB MAB phase into two-dimensional MoB MBenes with a good wrinkled structure. This material exhibited excellent electrochemical performance in sodium-ion batteries (SIBs), achieving a reversible specific capacity of 196.5 mAh/g at a current density of 50 mA/g (Figure 12a–c). Liu et al. [146] proposed a sodium storage strategy combining interface engineering with Na_2_S adsorption, vertically growing SnS@C nanosheets on MBenes. The MBenes-SnS@C anode exhibited a high capacity of 411 mAh/g at a current density of 1 A/g and maintained a capacity of 420 mAh/g after 100 cycles at a current density of 0.5 A/g (Figure 12d,e). The CV curves of the MBenes-SnS@C-2 electrode at a scan rate of 0.1–1 mV/s show good redox characteristics and minimal peak displacement, indicating low polarization and fast electrochemical kinetics (Figure 12f–h).

### 4.2. Performance Comparison: MBene vs. Other Anode Materials

The practical deployment of any electrode material depends not only on its ability to host Na ions but also on its electrochemical performance, including reversible capacity, rate capability, cyclability, and safety [136,147]. Compared with more conventional materials such as hard carbon, transition metal oxides, or MXenes, MBenes have begun to distinguish themselves with several noteworthy advantages [148,149,150].

For example, hard carbon is commonly used as a reference anode material in SIBs, offering reversible capacities of ~300 mAh/g and reasonable cycling stability [151,152,153]. However, its poor rate performance and limited electrical conductivity often restrict its application in fast-charging scenarios. MBene anodes have demonstrated higher intrinsic electrical conductivity, which enables rapid electron transfer and supports higher charge–discharge rates [154]. Laboratory reports reveal that certain MBene compositions (e.g., Mo_2_B_2_ or Ti_2_B_2_-based MBenes) can deliver initial reversible capacities between 350–500 mAh/g with excellent rate capability and retention of 80–90% of initial capacity after several hundred cycles under practical current densities [40,81].

Compared with MXenes, MBenes sometimes offer superior cycling stability given their stronger metal–boron bonds (as opposed to metal–carbon/nitrogen) that can better withstand repeated sodiation and desodiation [155,156,157]. While MXene anodes may suffer from rapid capacity fade due to structural fatigue, MBenes’ robust lattice provides resilience and supports more stable SEI formation [158]. Metal oxides, though they may promise high capacity, often experience significant irreversible capacity loss and poor rate capability due to volume expansion and sluggish Na ion diffusion, which are drawbacks that MBene architecture can help ameliorate [159].

Despite these advantages, MBene anodes are not without drawbacks. Their initial Coulombic efficiency can be somewhat lower than that of traditional hard carbons due to the formation of SEI or irreversible trapping of Na at defect or functional group sites [160]. Still, ongoing research on surface engineering and electrolyte optimization is addressing these limitations, gradually aligning MBenes’ practical and theoretical performance.

### 4.3. Sodium Storage and Migration Mechanisms in MBenes

A nuanced understanding of how Na ions are stored, migrate, and interact within host materials is essential for optimizing performance and predicting long-term durability. Na storage in MBene electrodes is widely recognized to follow a hybrid mechanism that encompasses intercalation between layers, pseudocapacitive surface adsorption, and, in certain cases, conversion reactions involving active transition metals. In SIBs, the charge storage mechanism of MBenes primarily relies on interlayer insertion reactions and surface adsorption/redox reactions. Na^+^ can be inserted into the interlayer gaps of MBenes, while the abundant transition metal sites on the surface (e.g., Mo, Ti, etc.) provide additional pseudocapacitive capacity through redox reactions. The layered structure and tunable interlayer spacing of MBenes help mitigate volume expansion during Na^+^ intercalation, while surface functional groups (e.g., –O, –F) may participate in ion adsorption, enhancing interfacial charge transfer.

Intercalation [161,162] is the primary Na storage mechanism in most MBenes, wherein Na ions insert into galleries between MBene layers during discharge. The process is largely reversible, especially when the interlayer spacing is optimized. In situ X-ray diffraction and atomic force microscopy have shown that sodiation leads to moderate, reversible expansion of the MBene lattice, in a process far less destructive than observed in many oxide or alloying-type anodes. The strong metal–boron bonds and the planar geometry of MBene flakes help contain the structural changes associated with sodiation and desodiation, preventing pulverization or exfoliation.

Regarding surface adsorption and pseudocapacitance [163,164], MBene sheets are typically terminated with surface functionalities such as –OH, –O, or –F due to synthesis protocols. These groups provide abundant high-energy adsorption sites for Na ions, leading to substantial pseudocapacitive contributions. Such surface-driven storage mechanisms enhance the high-rate performance and capacitance of MBene anodes, facilitating the fast-charging and discharging crucial for modern SIB applications.

In some cases, Na storage may involve partial conversion reactions [165,166], forming transient nanophases or alloys within the material during cycling, especially for MBenes containing redox-active transition metals. While these reactions can provide extra capacity, they must be carefully managed to prevent irreversible phase changes and capacity loss over long cycles.

Na ion migration through MBene electrodes is facilitated by both the intrinsic conductivity of the material and the accessible, low-energy diffusion pathways within and on the surfaces of the layers. Computational investigations using density functional theory have highlighted low Na ion diffusion barriers on MBene surfaces, often lower than for analogous carbon or oxide electrodes, thus confirming the theoretical advantages of MBene frameworks for SIB operation.

### 4.4. MBene-Based Composite Materials: Enhancing Sodium-Ion Battery Performance

To overcome remaining limitations such as layer stacking, agglomeration, and suboptimal Coulombic efficiency, numerous studies have explored the integration of MBenes with other functional materials to yield high-performance composites. These composite designs have often been inspired by analogous efforts in MXene and graphene research, leveraging synergies between the constituents for higher overall battery performance.

With regard to MBene/carbon composites [167,168], the incorporation of conductive carbon frameworks (e.g., graphene, carbon nanotubes, amorphous carbon) with MBene nanosheets is highly effective for preventing restacking, improving electrode flexibility, and promoting electron/ion transport. Such hybrid electrodes exhibit increased surface area, suppressed aggregation, and enhanced rate capability, enabling practical capacities and long-cycle durability under demanding conditions.

Polymer and metal oxide composites have been used [169,170]. Polymers, especially those with elastic or self-healing properties, can buffer volume changes during repeated sodiation/desodiation and improve structural robustness. Metal oxides, when homogeneously integrated with MBenes, may introduce additional Na storage sites while acting as spacers to keep the MBene layers apart, further enhancing cycling stability and rate performance.

For optimization of surface chemistry [171,172], surface engineering remains a key method for tuning Na affinity, controlling SEI formation, and improving first-cycle efficiency. Rational choice of composite type, composition ratio, and synthesis method—whether in situ assembly, physical mixing, or solution-phase hybridization—offers a powerful toolkit for tailoring MBene-based electrodes to the specific needs of SIBs technologies.

MBene materials have rapidly established themselves as among the most exciting 2D platforms for next-generation SIBs. Their unique structural adaptability, robust bonding, and outstanding electrical properties address several fundamental challenges facing this battery technology. However, to reach full industrial relevance, further research is needed to optimize synthesis, engineer surfaces and interfaces, and master the design of multifunctional composite electrodes. With continued innovation, MBene-based materials may soon enable production of high-performance, cost-effective, and long-lived SIBs with far-reaching impact for grid storage and beyond.

## 5. Challenges and Development Trends

**Figure 13:**
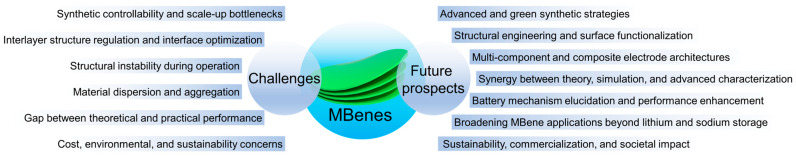
Schematic representation of current challenges and future prospects for MBenes.

The emergence of MBenes as novel 2D boride materials has created exciting opportunities to revolutionizing LIB and SIB technologies. Despite their unique structures and outstanding electrochemical properties, significant scientific and technological challenges remain before MBenes can be realistically implemented in commercial electrochemical storage systems. Addressing these issues is essential for translating the impressive laboratory results of MBenes into meaningful real-world battery advancements. In this section, we discuss the main challenges facing the application of MBenes in these batteries and highlight research trends aimed at overcoming them (Figure 13).

(I) Synthetic controllability and scale-up bottlenecks

A major obstacle hindering the widespread adoption of MBene-based electrodes is the challenge of achieving highly controllable, reproducible, and scalable synthetic methods. Most conventional approaches for producing MBenes, such as selective chemical etching and top-down exfoliation of bulk precursors, typically require harsh chemicals (e.g., strong acids or fluorides), stringent environmental controls, and labor-intensive procedures. Research has found that a 2 M LiF/6 M HCl solution causes corrosion of the Mo_2_B_2_ layer, while even orthogonal Mo_2_B_2_ cannot be completely etched out of MoAlB using a 10% NaOH solution, because low-concentration alkaline solutions can etch only the surface layer of the MAX phase [71,173]. These constraints lead to problems such as low yield, poor batch-to-batch consistency, and limited control over flake thickness, lateral size, defect density, and surface terminations. Scaling up for industrial application presents an even greater challenge, as processes must deliver high throughput, impurity control, and cost efficiency. Current research trends are addressing these bottlenecks by exploring greener synthetic protocols, such as molten salt methods, electrochemical exfoliation, and mechanochemical approaches that significantly reduce environmental hazards and operational complexity. Developing closed-loop, solvent-free, or recyclable processes aligns with global trends toward sustainable manufacturing. Additionally, optimization precursor selection, process automation, and standardizing process parameters are key directions for enabling large-scale production of MBenes with consistent quality, which is critical for battery manufacturing.

(II) Interlayer structure regulation and interface optimization

Another significant challenge involves fine-tuning the interlayer structure and optimizing interfaces within MBene materials, both of which are essential for efficient ion transport and stable electrochemical performance. While MBenes offer unique advantages including their layered architecture and high specific surface area, these features can also be problematic; uncontrolled stacking or aggregation diminishes ion accessibility and hinders charge transfer. To overcome the self-weight stacking problem and improve electrochemical performance, Li et al. [110] reported the use of CNTs in combination with a single-layer MBene (forming mono-MBene/CNT). This composite material effectively prevented the nanoplates from stacking due to their weight, thereby significantly improving the rate performance and cycling stability of the electrode material.

Additionally, the interlayer spacing in pristine MBenes may not be ideally suited to the ionic radii of Li or Na, particularly in SIBs, which require broader galleries to facilitate Na diffusion. Recent research has focused on modifying interlayer chemistry through approaches such as ion intercalation (e.g., inserting organic cations or small molecules), surface functionalization (e.g., with oxygen, hydroxyl, or other moieties), and the intentional introduction of defects and dopants. These modifications can expand interlayer spacing, improve wettability, and facilitate faster, more reversible ion migration. At the electrolyte interface, engineering the chemical environment (by modifying terminal groups or using protective coatings) can mitigate the formation of unstable SEI and suppress side reactions. The advent of in situ and operando characterization tools has also accelerated insights into how MBene structures evolve during battery operation, providing a sound foundation for interface and structure optimization.

(III) Structural instability during operation

Stable cycling at high charge–discharge rates and over many cycles is essential for any practical battery material. For MBenes, preserving mechanical and electrochemical integrity during extended use is challenging. Repeated ion insertion and extraction cause volumetric changes that, if too great, can result in structural distortion, particle fragmentation, nanosheet delamination, and even electrical disconnection within the electrode. Current strategies to address these issues include enhancing interlayer interactions via cross-linking, integrating flexible or elastic matrices, and designing MBene-based composite electrodes. Incorporation of carbon nanostructures, conductive polymers, or hybrid binders can alleviate mechanical stress, prevent sheet restacking, and help maintain continuous conductive networks. Additionally, optimization of the electrode architecture, including controlling porosity, flake orientation, and thickness, can better accommodate expansion and contraction, thereby maximizing cycle life without compromising capacity or rate performance.

(IV) Material dispersion and aggregation

Achieving molecular-scale dispersion of MBene nanosheets is essential for maximizing the active surface area and improving overall battery performance. However, MBenes naturally tend to agglomerate or restack due to strong van der Waals forces and high surface energy, resulting in reduced accessible surface, fewer active sites, and hindered ion transport. To address this, surface engineering and composite design strategies are increasingly being utilized. Surface functionalization with charged groups, incorporation of spacers (such as organic molecules or nanoparticles), and integration with dispersive matrices like graphene or conductive polymers have all demonstrated effectiveness in preventing aggregation. Furthermore, in situ exfoliation methods, where MBenes are generated and processed in a single step or within a composite, can improve processability and ensure uniform dispersion of electroactive components throughout the electrode.

(V) Gap between theoretical and practical performance

Although computational studies and small-scale laboratory tests highlight MBenes’ remarkable theoretical properties, such as high capacity and exceptional conductivity, the translation of these findings to commercial-level performance has often lagged behind. Several factors contribute to this gap; real-world electrodes are typically thicker, polydisperse, and incorporate binders or conductive additives, all of which can hinder rapid ion transport, electron conduction, and mechanical stability. Additionally, parasitic side reactions and imperfect interfaces further diminish effective utilization of materials. Bridging this divide requires close feedback between theory, modeling, and practical engineering. Increasingly sophisticated multi-scale simulations (DFT, molecular dynamics, continuum models) are now being intergrated with high-fidelity experimental validations to refine both material design and cell assembly protocols. Furthermore, the adoption of advanced operando diagnostics provides richer datasets to elucidate failure modes, capacity fade, and real-world performance limitations. Interdisciplinary collaboration among theoretical chemists, material scientists, and process engineers is essential to align predictions and outcomes for next-generation battery design.

(VI) Cost, environmental, and sustainability concerns

The main technical bottlenecks hindering the transformation of MBenes from laboratory-scale demonstration to commercial battery technology lie in the reproducibility and cost issues during large-scale production. Currently, the synthesis process of MBenes is complex and energy-consuming, leading to high production costs and making it difficult to achieve economic feasibility in mass production. As the main precursor, the cost of MAB varies depending on the type of transition metal contained, its purity, and market supply-demand relationships. If rare or expensive transition metals are involved, the cost may be relatively high. In addition, the long-term stability and cycle life of MBenes still need further optimization, especially in terms of their durability performance under actual usage environments. For industrial applications, balancing high performance with low costs, improving production efficiency, and ensuring material consistency and reliability are the key technical challenges faced by this field. Although certain progress has been made in laboratory synthesis methods, the further development of efficient, reproducible, and precisely controlled nanoscale synthesis technologies is required to achieve industrial-scale production. Meanwhile, the establishment of recycling pathways is also crucial. Waste materials, defective products, and other by-products generated during the production process need effective recycling treatment to improve resource utilization, reduce costs, and minimize environmental pollution.

In summary, the application of MBenes in LIBs and SIBs holds significant promise, but realizing this potential will require focused research to overcome synthetic, structural, interfacial, and practical engineering challenges. The ongoing integration of advanced synthetic chemistry, surface engineering, theory-driven design, and sustainable manufacturing is expected to accelerate the transition from laboratory to industry, ensuring that MBenes make a meaningful contribution to the future of clean energy storage. Emerging solutions and research directions not only address current limitations but also pave the way for technological breakthroughs in high-performance, durable, and sustainable battery systems.

## 6. Future Prospects

As the global pursuit of sustainable, efficient, and high-capacity energy storage solutions intensifies, the development and optimization of advanced materials such as MBenes are expected to play a pivotal role in shaping the next generation of batteries. While significant progress has been made in understanding and applying these novel 2D boride materials in LIBs and SIBs, numerous opportunities and challenges persist for both fundamental research and technological implementation. Looking forward, several strategic directions are set to unlock the full potential of MBenes, ranging from innovative synthesis methods and theoretical investigations to hybrid material design, mechanistic insights, and expanded application prospects (Figure 13).

(I) Advanced and green synthetic strategies

Efficient and scalable synthesis remains fundamental to the widespread adoption of any new material. For MBenes, particular attention must be given to advancing synthetic methods that can reliably produce high-quality, defect-controlled nanosheets while meeting environmental and industrial standards. Conventional synthesis techniques, such as chemical etching or top-down exfoliation, though effective, often involve hazardous chemicals, yield low quantities, or offer limited control over layer thickness and surface composition. The transition toward green synthesis and environmentally friendly protocols is both a scientific necessity and an industrial priority. Alternative approaches may include mechanochemical synthesis, electrochemical exfoliation, and bio-inspired methods that reduce energy consumption and environmental impact. Additionally, fine-tuning synthetic parameters—such as temperature, precursor concentration, and reaction time—will be essential for producing MBenes with uniform size, desirable surface functionality, and minimal defects. The development of closed-loop, recyclable, and energy-efficient synthesis platforms will not only minimize waste but also lower production costs, thereby accelerating commercial deployment.

(II) Structural engineering and surface functionalization

Fully harnessing the exceptional properties of MBenes demands ongoing advancements in material engineering at the atomic and molecular scales. Rationally designing the interlayer spacing, through ion exchange, organic molecule intercalation, or targeted doping, offers a pathway to optimize ion mobility and accommodate the significant volume changes induced by charge–discharge cycles. These approaches are particularly crucial for SIBs, where the larger ionic radius presents greater challenges than in Li systems. Surface functionalization represents another essential direction. The intentional introduction, modification, or removal of terminal groups—such as hydroxyl, oxygen, or fluorine—can tailor the chemical reactivity, electronic structure, and interfacial behavior of MBenes. By tuning these surface characteristics, it becomes possible to optimize electrode-electrolyte compatibility, enhance solid electrolyte interphase stability, and suppress parasitic side reactions that compromise long-term cycling. Notably, controlled surface modifications may also render MBenes suitable for multifunctional hybrid systems, enabling the exploitation of synergistic properties across different material classes.

(III) Multi-component and composite electrode architectures

Looking beyond the use of pristine MBene nanosheets, future research will increasingly emphasize the development of multi-component composite materials that integrate MBenes with other advanced functional phases. For instance, MBene–carbon, MBene–polymer, or MBene–metal oxide composites have demonstrated significant potential for achieving high conductivity, enhanced mechanical strength, and diverse charge–storage mechanisms simultaneously. Through the rational design of heterostructures or hybrid electrodes, scientists can further improve rate capabilities, mitigate structural degradation, and preserve electrolyte accessibility. Moreover, incorporating flexible or self-healing polymers could enable applications in flexible and wearable electronics, while strategic combinations with metal oxides may facilitate dual intercalation/conversion-type storage for exceptional capacity gains. Leveraging synergistic effects through composite engineering will therefore be central for unlocking the practical benefits of MBenes in next-generation battery technologies.

(IV) Synergy between theory, simulation, and advanced characterization

The convergence of theoretical modeling, computational screening, and advanced in situ characterization techniques is poised to transform the understanding and optimization of MBene-based systems. Atomistic simulations, such as density functional theory and molecular dynamics, will play a crucial role in predicting optimal compositions, surface configurations, and reaction pathways, thereby guiding targeted synthesis and property modulation. These theoretical frameworks, when combined with high-throughput screening methods, can dramatically accelerate material discovery and customization. Experimentally, the application of in situ and operando techniques—including synchrotron magnetic, spectroscopic, and X-ray synchrotron radiation—will illuminate the dynamic evolution of MBenes’ structure, interfaces, and chemical environments during real-time battery operation. Achieving a deeper mechanistic understanding of Li and Na storage, degradation, and interfacial reactions will enable more precise material and device engineering. Therefore, the integration of theory and experiment remains fundamentals to advancing next-generation MBene research.

(V) Elucidating battery mechanisms and performance enhancement

One of the most promising areas for future research is the ongoing elucidation of ion storage mechanisms in MBenes. Whether through classical intercalation, pseudocapacitive surface storage, or conversion reactions, unraveling these processes at the atomic level will guide the rational design of materials with tailored performance characteristics. Special attention must be given to balancing high energy and power densities with exceptional cycling stability, a necessity for electric vehicles and large-scale stationary storage. Additionally, active control of the SEI remains a critical issue, as SEI stability governs Coulombic efficiency and cell longevity. Investigating MBene–electrolyte interactions, identifying additives that stabilize the interphase, or engineering artificial SEIs could provide pathways to reliable, high-performance batteries.

(VI) Broadening MBene applications beyond lithium and sodium storage

While LIBs and SIBs represent the immediate frontiers of MBene research, the unique properties of these materials suggest far broader application possibilities. For instance, MBenes could serve key roles in aqueous batteries, magnesium/zinc-ion batteries, supercapacitors, electrocatalysis, and sensors, owing to their excellent electrical conductivity, chemical tunability, and mechanical resilience. Additionally, the high surface area and chemical reactivity of MBenes make them promising candidates for environmental remediation, water splitting, and hydrogen storage. Harnessing these functionalities while drawing insights from battery-focused research will foster cross-disciplinary progress and drive the development of multifunctional MBene-based devices.

(VII) Sustainability, commercialization, and societal impact

For real-world application, MBene research must increasingly focus on environmental sustainability and economic feasibility. This involves not only green synthesis but also the recyclability of MBene-containing electrodes and their integration into existing manufacturing ecosystems. Evaluating resource availability, lifecycle impacts, and end-of-life scenarios should inform industrial scaling strategies. Finally, fostering collaboration among academia, industry, and policymakers will be crucial to translating MBene science into tangible societal benefits, from affordable, high-performance batteries that support renewable energy grids to safer, longer-lasting batteries for consumer devices and transportation.

MBenes, as an emerging family of two-dimensional transition metal borides, boast core advantages such as tunable layered structures, high electrical conductivity, and excellent electrochemical stability. In LIBs and SIBs, MBenes demonstrate exceptional capacity, long-term cycling durability, and high-rate performance. Defect engineering and composite strategies further enhance their practicality. However, key challenges remain, including scalable and precise synthesis methods, improvements in thermodynamic stability, and environmentally friendly processing techniques. Looking ahead, integrating sustainable manufacturing, achieving atomic-level structural design through computational modeling, and expanding into hybrid energy platforms represent promising research directions. By combining fundamental materials science with engineering considerations, MBenes holds the potential to drive the development of next-generation electrochemical energy storage systems, achieving higher energy density and operational reliability, and ultimately contributing to the global transition toward sustainable energy solutions.

## Figures and Tables

**Table 1:** Capacity and diffusion energy of several 2D materials.

Materials	Types	Capacity (mA h/g)	Diffusion Energy (eV)	Refs.
Mo_2_B_2_	LIBs	444	0.270	[23]
TiB	LIBs	480	0.020	[24]
	SIBs	480	0.020	[24]
Ti_2_B_2_	LIBs	456	0.017	[25]
	SIBs	342	0.008	[25]
Ti_3_C_2_	LIBs	320	0.280	[26]
	SIBs	370	—	[27]
Ti_2_C	SIBs	359	—	[28]
Graphene	LIBs	372	0.400	[29]
MoS_2_	SIBs	669	0.460	[30,31]
Black phosphorus	LIBs	2596	0.080	[32]
Monolayer h-BN	LIBs	762	0.100	[33,34]
	SIBs	571	—	[33]
g-C_3_N_4_	LIBs	1166	0.570	[35,36]

## Data Availability

Source data are available from the corresponding authors upon reasonable request.
